# Mitochondrial Mutations in Ethambutol-Induced Optic Neuropathy

**DOI:** 10.3389/fcell.2021.754676

**Published:** 2021-10-05

**Authors:** Xiao-Hui Zhang, Yue Xie, Quan-Gang Xu, Kai Cao, Ke Xu, Zi-Bing Jin, Yang Li, Shi-Hui Wei

**Affiliations:** ^1^Department of Ophthalmology, The Chinese People’s Liberation Army General Hospital, The Chinese People’s Liberation Army Medical School, Beijing, China; ^2^Beijing Ophthalmology and Visual Sciences Key Laboratory, Beijing Institute of Ophthalmology, Beijing Tongren Eye Center, Beijing Tongren Hospital, Capital Medical University, Beijing, China

**Keywords:** ethambutol, optic neuropathy, *OPA1*, mitochondrial DNA, mutation

## Abstract

**Background:** Ethambutol-induced optic neuropathy (EON) is a well-recognized ocular complication in patients who take ethambutol as a tuberculosis treatment. The aim of the current study was to investigate the presence of mitochondrial mutations, including *OPA1* and Leber’s hereditary optic neuropathy (LHON)-mitochondrial DNA (mtDNA), in patients with EON and to determine their effect on clinical features of these patients.

**Methods:** All 47 patients underwent clinical evaluations, including best-corrected visual acuity, fundus examination, and color fundus photography; 37 patients were then followed up over time. Molecular screening methods, including PCR-based sequencing of the *OPA1* gene and LHON-mtDNA mutations, together with targeted exome sequencing, were used to detect mutations.

**Results:** We detected 15 *OPA1* mutations in 18 patients and two LHON-mtDNA mutations in four patients, for an overall mutation detection rate of 46.8%. The mean presentation age was significantly younger in the patients with the mitochondrial mutations (27.5 years) than in those without mutations (48 years). Fundus examination revealed a greater prevalence of optic disc hyperemia in the patients with mutations (70.5%) than without mutations (48%). Half of the patients with mutations and 91% of the patients without mutations had improved vision. After adjusting for confounders, the logistic regression revealed that the patients with optic disc pallor on the first visit (*p* = 0.004) or the patients with the mitochondrial mutations (*p* < 0.001) had a poorer vision prognosis.

**Conclusion:** Our results indicated that carriers with *OPA1* mutations might be more vulnerable for the toxicity of EMB to develop EON.

## Introduction

Ethambutol (EMB), one of the first-line drugs used to treat tuberculosis, works by inhibiting the arabinosyl transferase of mycobacteria (Belanger et al., 1996). However, its use is associated with a well-recognized ocular complication, ethambutol-induced optic neuropathy (EON), which is characterized by blurring of the vision, dyschromatopsia, and central or cecocentral scotoma (Carr and Henkind, 1962). The ocular symptoms usually appear after 7 to 8 months of treatment with EMB, and the development of EON is both time- and dose-dependent (Fraunfelder et al., 2006; Lee et al., 2008). The incidence of EON is about 1% in patients prescribed an EMB dose at 15 mg/kg/d (Santaella and Fraunfelder, 2007; Yang et al., 2016). The risk factors for the occurrence of EON include older age, low body weight, and renal dysfunction (Chen et al., 2012). About 30 to 54% of the patients have varying degrees of vision recovery at 3 to 7 months after EMB discontinuation (Lee et al., 2008; Ezer et al., 2013); however, some patients have severe and permanent visual loss, even without the known risk factors. Therefore, other predisposing factors might exist for EON.

At present, the pathophysiology of EON still remains unclear. Several previous studies have indicated that EMB disrupts energy metabolism and the network structure of mitochondria by inducing severe vacuole formation and by decreasing membrane potential (Chung et al., 2009; Guillet et al., 2010). Dotti et al. (1998) first described the m.G11778A mitochondrial DNA (mtDNA) mutation in an EON patient, pointing to a relationship between mitochondrial mutation and the development of EON. Leber’s hereditary optic neuropathy (LHON) is caused by mtDNA mutations that disrupt complex I activity of the mitochondrial respiratory chain and ATP synthesis (Zhang et al., 2018). Autosomal dominant optic atrophy (ADOA) is another form of mitochondrial optic neuropathy that is caused by mutations of the *OPA1* gene (Delettre et al., 2000). The *OPA1* gene encodes a dynamin-related GTPase, which is localized at the inner membrane of mitochondria and plays a role in mitochondrial fusion (Kao et al., 2015).

Both LHON and ADOA have a common pathophysiological outcome, which is mitochondrial energy deficiency and retinal ganglion cell apoptosis (Kao et al., 2015; Zhang et al., 2018). The current literature reports 8 patients carrying LHON mtDNA mutations and 2 patients harboring *OPA1* mutations who developed optic neuropathy during the use of EMB (Dotti et al., 1998; De Marinis, 2001; Hwang et al., 2003; Chowdhury et al., 2006; Ikeda et al., 2006; Guillet et al., 2010; Pradhan et al., 2010; Seo et al., 2010). Most of these studies are case reports, and the final clinical diagnosis is controversial for those patients. The relationship between the mitochondrial mutations and the clinical features of EON has not been fully studied.

In the present study, we investigated the presence of mitochondrial mutations (*OPA1* and LHON-mtDNA) in a cohort of 47 patients with EON. We also studied the effects of these mutations on the clinical features of the patients by performing genetic analysis and identified disease-causing gene mutations in 22 patients.

## Materials and Methods

### Patients

This study was approved by the Medical Ethics Committee of Beijing Tongren Hospital. All investigations followed the tenets of the Declaration of Helsinki. Clinical data were retrospectively collected from outpatient and hospitalized patients diagnosed with EON from 2011 to 2018 at the Department of Ophthalmology in Chinese People’s Liberation Army General Hospital and at the Genetics Laboratory of the Beijing Institute of Ophthalmology at Beijing Tongren Hospital. Blood was taken at initial presentation for genetic analysis with the patients’ or their parents’/guardians’ consent. We recruited a total of 47 unrelated patients, and 37 patients were followed up either by revisit evaluation (two patients) or by telephone surveys (35 patients). All patients underwent ophthalmological evaluations, including the best corrected visual acuity (BCVA), slit-lamp biomicroscopy, fundus examination, and color fundus photography. Some patients had color perception tests (Lanthony 15-Hue, Farnsworth D-15, or Ishihara color plates), Octopus or Humphry visual field tests, and spectral domain OCT examinations. We extracted genomic DNA from peripheral blood leukocytes from all patients and from available family members, following the manufacturer’s instructions (Vigorous, Beijing, China).

The inclusion criteria for EON were based on previous guidelines (Fraunfelder et al., 2006; Lee et al., 2008). The diagnosis had to satisfy the major criterion and at least two of the minor criteria. The major criterion is vision loss appearing only after taking EMB and within 2 months after discontinuation of the drug. The minor criteria are: (1) abnormal results on color perception tests, and (2) central, paracentral, or cecocentral scotoma or temporal hemianopia on visual field examinations, and (3) optic disc hyperemia or pallor on color fundus photography. Exclusion criteria include optic neuritis, glaucoma, and other retinal diseases.

### PCR-Based Sequencing of the *OPA1* Gene and Leber’s Hereditary Optic Neuropathy-mtDNA

All coding regions of the *OPA1* gene and 19 primary LHON-mtDNA mutations were amplified by PCR in 36 patients. The primer sequences and the product lengths for amplification were described previously (Chen et al., 2014). The 19 primary LHON-mtDNA mutations included m.11778G > A, m.3460G > A, m.14484T > C, m.3376G > A, m.3635G > A, m.3697G > A, m.3700G > A, m.3733G > A, m.4171C > A, m.10197G > A, m.10663T > C, m.13051G > A, m.13094T > C, m.14459G > A, m.14482C > A, m.14482C > G, m.14495A > G, m.14502T > C, and m.14568C > T^1^. Purified PCR products were sequenced with an ABI PRISM 3730 DNA sequencer (Applied Biosystems, Foster City, CA, United States). Sequencing data were compared with the GenBank sequence for the *OPA1* gene (NM_015560) and mtDNA sequence (AC_000021.2).

### Targeted Exome Sequencing

Eleven patients were investigated by TES with a capture panel including 194 known neuro-ophthalmological genes (Supplementary Table 1). The Illumina library preparation and capture experiments were performed as previously reported (Sun et al., 2018). Briefly, genomic DNA was fragmented by endonuclease digestion and used to capture the targeted genomic sequences. The amplicon-based enrichment library was sequenced on an Illumina NextSeq 500 (Illumina, Inc., San Diego, CA, United States). After removing the sequencing adapters, low quality reads, and duplicated reads, the high quality reads were aligned with the reference human genome (hg19) using the Burrows-Wheeler Aligner. Single nucleotide polymorphisms and insertions or deletions were called using the Genomic Analysis Toolkit Haplotype Caller.

### Bioinformatics Analysis

The potential functional impacts of missense mutations were evaluated with Polyphen-2^2^, Mutation Taster^3^, and SIFT^4^. The effect of splicing mutations was analyzed with NetGene2^5^ and BDGP^6^. The allele frequency of the variants was confirmed in the 1,000 Genome Project^7^ and ExAC^8^. Co-segregation analysis was performed in available family members to verify the suspected mutations. We classified the variants into pathogenic, likely pathogenic, uncertain of significant, likely benign, and benign according to the guidelines published by the American Academy of Medical Genetics and Genomics (ACMG) (Richards et al., 2015).

### Statistical Analysis

We converted the Snellen ratios into logarithm of the minimum angle of resolution (logMAR) values for statistical purpose. LogMAR values of 0, 1.0, and 2.0 are equal to a Snellen vision of 1.0, 0.1, and counting fingers, respectively (Holladay, 1997). The Wilcoxon rank sum test and Pearson Chi-square or Fisher’s exact test were used to analyze the quantitative and categorical data, respectively. The Kruskal–Wallis test or multivariate logistic regression was used to analyze correlations. We performed all statistical analysis using SPSS version 22 software (IBM Corporation, New York, United States). The statistical significance level was 5%.

## Results

### Mitochondrial Mutation Detection Rate and Related Mutations

We detected *OPA1* and LHON-mtDNA mutations in 22 of the 47 patients with EON, for an overall mutation detection rate of 46.8%. Mutations in 15 patients were detected by Sanger sequencing and mutations in 7 patients were detected by TES (Supplementary Table 2). The average coverage of the TES was 99.8%. The average sequencing depth was 288X. About 99% of the data had a depth of 10X or more.

We identified 15 distinct mutations of the *OPA1* gene in 18 patients (Supplementary Figure 1), for a detection rate of 38.3%. According to the ACMG guidelines, 13 mutations were defined as pathogenic and two mutations were defined as likely pathogenic (Table 1). Of these mutations, 8 were newly detected in the current study. These mutations included five (33.3%) nonsense, four (26.5%) frameshift indels, three (20%) splicing defects, two (13.3%) missense, and one (7%) large deletion mutation (Table 1). The most common mutation was c.2708_2711delTTAG (p.V903Gfs^∗^3), with an allele frequency of 22.2% (4/18); the remaining 14 mutations were detected only once. The eight novel mutations included three frameshift indels, two missense, one splicing effect, one nonsense, and one large deletion. None of these novel mutations were observed in the public databases. The two missense mutations were predicted as disease-causing by three *in silico* analysis programs (Table 1).

**TABLE 1:** Fifteen mutations of the *OPA1* gene identified in this study.

**cDNA change**	**Protein change**	**Protein domain**	**Allele number**	**Polyphen-2**	**Mutation Taster**	**SIFT**	**PROVEAN**	**NetGene2**	**BDGP**	**1000G**	**ExAC**	**gnomAD**	**References**	**ACMG**
c.32 + 1G > C	-	basic	1		DC			donor loss	donor loss	N	N	N	This study	P
c.154C > T	p.R52X	basic	1		DC					N	N	4.02E-06	Ban et al., 2007	P
c.544_545 insT	p.T184Yfs*11	-	1		DC					N	N	N	This study	P
c.934C > T	p.R312X	GTPase	1		DC					N	N	N	Cardaioli et al., 2006	P
exon12-17 deletion	-	GTPase, dynamin	1		-					N	N	N	This study	P
c.1516 + 1G > A	-	GTPase	1		DC			donor loss	donor loss	N	N	N	Santarelli et al., 2015	P
c.1631T > A	p.L544X	dynamin	1		DC					N	N	N	This study	P
c.1847 + 1_ + 4del	-	dynamin	1		DC			donor loss	donor loss	N	N	N	Ferré et al., 2009	P
c.1808A > C	p.D603A	dynamin	1	PD	DC	D	Deleterious	no splice sites change	no splice sites change	N	N	N	This study	LP
c.2111T > A	p.V704D	dynamin	1	PD	DC	D	Deleterious	no splice sites change	no splice sites change	N	N	N	This study	LP
c.2121_2124del	p.S708Lfs*14	dynamin	1		DC					N	N	N	This study	P
c.2131C > T	p.R711X	dynamin	1		DC					N	N	N	Delettre et al., 2001	P
c.2197C > T	p.R733X	dynamin	1		DC					N	N	N	Li et al., 2008	P
c.2246_2247insTTA	p.E749Dfs*2	dynamin	1		DC					N	N	N	This study	P
c.2708_2711delTTAG	p.V903Gfs*3	GTPase effector	4		DC					N	N	N	Delettre et al., 2000	P

*PD, probably damaging; DC, disease causing; D, damaging; N, none; P, pathogenic; LP, likely pathogenic.*

We detected the m.11778G > A mutation in two patients and the m.14484T > C mutation in two patients. The detection rate of LHON-mtDNA mutations was 8.5%.

### Demographics and Clinical Characteristics of All Patients

The 47 patients in the current study included 31 males and 16 females, for a male-to-female ratio of 1.9:1 (Supplementary Table 2). Of these patients, seven had a family history of optic neuropathy, two carrying an *OPA1* mutation and four harboring an mtDNA mutation. The mean age of presentation was 39 years (range 16–74 years). The mean daily dose of EMB was 12.9 mg/kg (range 3.8–29.2 mg/kg) and the mean medication duration was 4 months (range 1–24 months). All patients had a major complaint of blurred vision, and 41 (87.2%) of them experienced the visual impairment simultaneously in both eyes. The mean course of the disease, defined as the time interval from the vision loss to the first visit, was 2 months (range 0.5–24 months). Eight patients had a complaint of ocular pain, numbness of a lower extremity, or hearing loss. Their mean BCVA was 1.0 (logMAR; range 0.5–1.3), which was not correlated with their daily EMB dose (*p* = 0.646), their medication duration (*p* = 0.099), or the course of their disease (*p* = 0.939).

**FIGURE 1:**
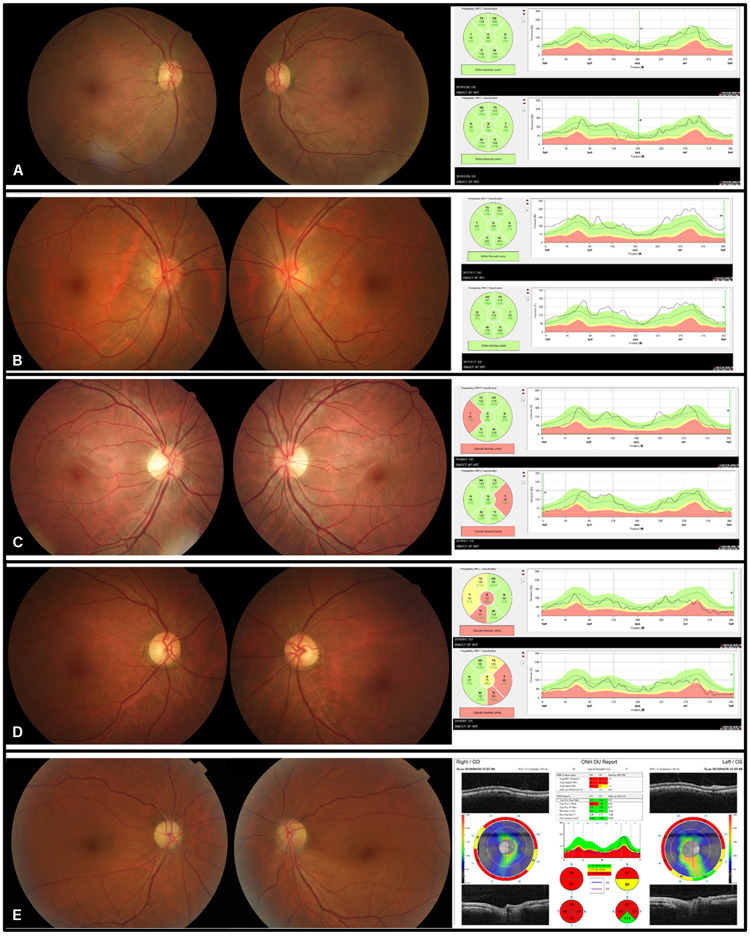
Five kinds of disc appearances and OCT images in five patients with EON. **(A)** The disc appearance and the RNFL thickness were normal. **(B)** The optic discs were hyperemic. The superior, temporal, and inferior RNFL was thickened. **(C)** The border of the nasal discs was blurred and the temporal discs were pale. The temporal RNFL thickness was thinner than normal. **(D)** The temporal discs were pale and the temporal RNFL became thin. **(E)** The optic discs were pale, with the inferior border of the disc blurred in the left eye.

**FIGURE 2:**
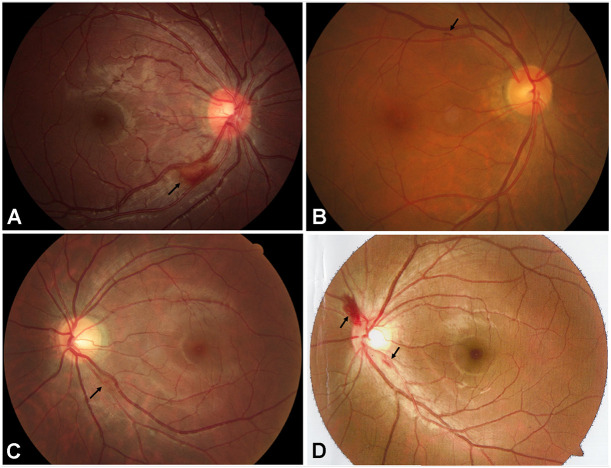
Fundus appearance of 4 patients with retinal hemorrhage (black arrows). **(A)** Retinal hemorrhage located at the inferior temporal side of the optic disc in the right eye of the patient A781. **(B)** Retinal hemorrhage located at the superior temporal side of the optic disc in the right eye of the patient A2579. **(C)** Retinal hemorrhage located at the inferior temporal side of the optic disc in the left eye of the patient A3198. **(D)** Retinal hemorrhages located at the border of the optic disc in the left eye of the patient E004.

Fundus examination revealed a symmetrical fundus appearance in 43 patients (91.5%). Of the 94 eyes, 46 (48.9%) showed optic disc hyperemia and retinal nerve fiber layer (RNFL) swelling, 15 (16.0%) had temporal disc pallor, nine (9.6%) presented with nasal disc hyperemia and temporal disc pallor, four (4.2%) showed total disc pallor, and 20 (21.3%) presented a normal optic disc appearance (Figure 1). Unilateral retinal hemorrhage was observed in four patients (Figure 2). The result of logistic regression showed that the patients with optic disc pallor had a longer course of disease compared with patients with optic disc hyperemia or with normal optic disc appearance (*p* < 0.001).

Of the 33 patients (61 eyes) who had a color perception test, 23 eyes (37.7%) presented with red/green color vision impairment, 22 eyes (36.1%) showed total color blindness, eight eyes (13.1%) presented with yellow/blue color vision impairment, and eight eyes (13.1%) showed normal color perception. Of the 33 patients (66 eyes) who had a visual field examination, 31 eyes (47%) presented with central scotoma, 23 eyes (34.8%) showed cecocentral scotoma, six eyes (9.1%) presented with paracentral scotoma, and six eyes (9.1%) showed temporal hemianopia.

**FIGURE 3:**
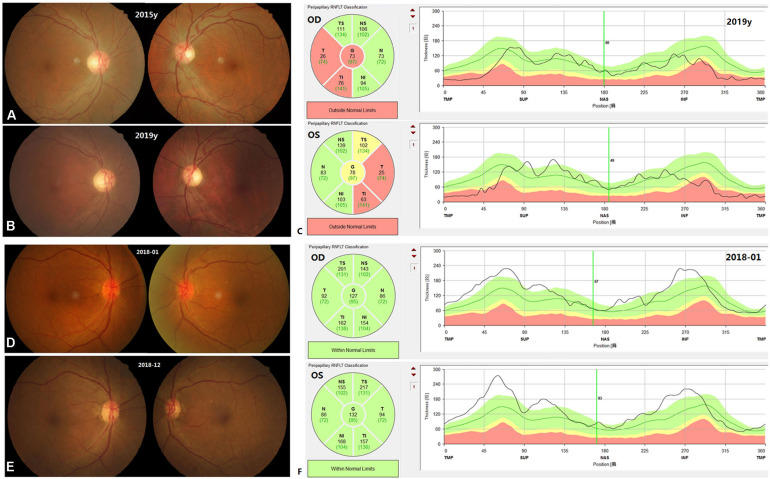
Fundus appearance and OCT images on the first visit and at follow-up for patient E005 **(A–C)** and patient A3124 **(D–F)**. In the patient E005, the fundus appearance did not change. The temporal RNFL in both eyes was below the normal limits at follow-up **(C)**. In the patient A3124, the color and border of the optic discs turned normal. The RNFL was thickened in both eyes on the first visit **(F)**.

The 37 patients were followed up over time (range 1–87 months), with a mean follow-up time of 15 months. Patient E005, carrying *OPA1* mutation p.R52X, and patient A3124, without mutations, underwent revisit examinations. After 4 years of follow-up, the BCVA of patient E005 improved from 0.02 in the right eye and 0.04 in the left eye to 0.1 in both eyes. The RNFL thickness at the temporal side of binocular optic discs was below the limits (Figure 3). After 11 months of follow-up, the BCVA of patient A3124 improved from 0.05 in the right eye and 0.03 in the left eye to 0.5 and 0.3, respectively. Fundus photography showed the optic disc hyperemia resolved at follow-up in both eyes (Figure 3). Of the remaining patients, 26 patients stated their VA had improved, eight patients reported their VA was unchanged, and one patient said his VA had worsened. None of patients with VA improved underwent cataract surgery or other intervention that would affect the assessment of visual outcome. The mean recovery time of the VA was 4 months (range 1–12 months). After adjusting for any confounders, the logistic regression revealed that the patients with optic disc pallor on the first visit (*p* = 0.004) or the patients with the mitochondrial mutations (*p* < 0.001) had a poorer vision prognosis (Supplementary Table 3).

### Comparison of Patients With Mitochondrial Mutations and Without Mutations

To simplify the description and statistical analysis, we divided the 47 patients into two groups: group 1 consisted of the patients carrying mitochondrial mutations and group 2 included the patients without mitochondrial mutations. We compared the difference in the demographics, EMB medications (Table 2), and clinical characteristics (Table 3) between the patients in the two groups. The mean visiting age was significantly younger in group 1 (27.5 years) than in group 2 (48 years). The percentage of patients with a family history was statistically higher in group 1 (27%) than in group 2 (4%). The percentage of eyes with optic disc hyperemia was higher in group 1 than in group 2. The visual outcome was better for group 2 than for group 1. Among the nine patients whose VA did not improve, six patients (66.7%) carried *OPA1* mutations and one patient (11.1%) carried the m.G11778A mutation.

**TABLE 2:** Comparison of the demographic and the EMB medication of the two groups of patients.

	**Group 1**	**Group 2**	***P* value**
No. of patients	22	25	–
*OPA1* mutant allele, *N* (%)	18 (81.8)	0	–
MtDNA mutant allele, *N* (%)	4 (18.2)	0	–
Male, N (%)	17 (77.27)	14 (56.00)	0.125‡
Mean age at presentation, years (range)	27.50 (16.00–73.00)	48.00 (17.00–74.00)	0.006†
Family history, *N* (%)	6 (27.27)	1 (4.00)	0.025‡
Smoking, *N* (%)	3 (15.00)	2 (8.00)	0.458‡
Renal dysfunction, *N* (%)	0 (0.00)	2 (9.52)	0.179§
Mean daily dose, mg/kg/d (range)	12.90 (3.80–16.90)	12.90 (8.80–29.20)	0.943†
Mean medication time, months (range)	3.75 (1.40–22.00)	5.00 (1.00–24.00)	0.864†

*Group 1, patients with mitochondrial mutations; Group 2, patients without mitochondrial mutations; ‡Pearson χ2 test; † Wilcoxon rank sum test; § Fisher’s exact test.*

**TABLE 3:** Comparison of the clinical characteristic of the two groups of patients.

	**Group 1**	**Group 2**	***P* value**
Mean LogMAR BCVA (range)	1.22 (0.10–1.70)	1.00 (0.10–2.00)	0.453†
Mean course of the vision loss, months (range)	2.00 (0.50–10.00)	2.00 (0.50–24.00)	0.497†
Disc appearance, eyes (%)	44 (100.00)	50 (100.00)	0.020‡
Normal	4 (9.09)	16 (32.00)	
Hyperemia	31 (70.45)	24 (48.00)	
Pallor	9 (20.46)	10 (20.00)	
Color vision, eyes (%)	24 (100.00)	37 (100.00)	0.607‡
Normal	3 (12.50)	5 (13.51)	
Red/green color vision impairment	7 (29.17)	16 (43.24)	
Yellow/blue color vision impairment	3 (12.50)	5 (13.51)	
Total color blindness	11 (45.83)	11 (29.74)	
Visual field, eyes (%)	34 (100.00)	32 (100.00)	0.059§
Central scotoma	18 (52.94)	13 (40.63)	
Paracentral scotoma	4 (11.76)	2 (6.25)	
Cecocentral scotoma	12 (35.30)	11 (34.38)	
Temporal hemianopia	0 (0.00)	6 (18.74)	
Vision outcomes, patients (%)	14 (100.00)	23 (100.00)	0.016‡
Improved	7 (50.00)	21 (91.30)	
Unchanged	6 (42.86)	2 (8.70)	
Decreased	1 (7.14)	0 (0.00)	
Mean recovery time, months (range)	6.00 (2.00–12.00)	3.00 (1.00–12.00)	0.107†

*Group 1, patients with mitochondrial mutations; Group 2, patients without mitochondrial mutations; †Wilcoxon rank sum test; ‡Pearson χ2 test; §Fisher’s exact test.*

## Discussion

The current study investigated the mitochondrial mutations in 47 unrelated patients with EON and described the clinical characteristics of the patients. We identified *OPA1* mutations in 18 (38.3%) patients and LHON-mtDNA mutations in four (8.5%) patients. EON is a dose-dependent form of toxic neuropathy related to several other risk factors, such as renal dysfunction and older age (Santaella and Fraunfelder, 2007; Chen et al., 2012; Yang et al., 2016). In the current cohort, 91.5% of the patients with EON had taken low-dose EMB (≤ 15mg/kg/day), 95.7% of the patients had a normal renal function, and 89.4% of the patients were younger than 60 years; therefore, the risk of developing ocular toxicity was relatively low in our cohort. Our results suggested that mitochondrial genetic variations, and especially *OPA1* mutations, are major predisposing factors for the occurrence of toxic optic neuropathy.

Mutations of the *OPA1* gene are responsible for 50–70% of ADOA, (Cohn et al., 2007; Yu-Wai-Man et al., 2010) which is the most common form of inherited optic neuropathy. *OPA1*-related ADOA is usually a mild and slowly progressive disorder (Cohn et al., 2007). Typically, patients suffer an insidious, symmetrical, and progressive visual defect in their childhood; however, the severity of visual impairment is highly variable (Cohn et al., 2007). About 10–20% of mutation carriers are “asymptomatic,” as they have a normal visual acuity or only a subtle visual disturbance (Cohn et al., 2007; Yu-Wai-Man et al., 2010). In the current cohort, we identified different kinds of mutations of the *OPA1* gene, but the type and location of these mutations were similar to those reported in typical patients with ADOA, (Cohn et al., 2007; Ferré et al., 2009; Yu-Wai-Man et al., 2010; Chen et al., 2014) except for the low frequency (13%, 2/15) of missense mutations. This frequency was only the half rate observed previously in patients with ADOA (approximately 27%) (Chen et al., 2014). Another previous study indicated that vision loss was usually more severe in patients with missense mutations than with null mutations (Yu-Wai-Man et al., 2010). Unlike the typical patients with ADOA, the *OPA1* mutation carriers in the current study all experienced a subacute visual loss while taking EMB therapy, and none of them noticed a visual abnormality before the treatment; therefore, they might be “asymptomatic” cases. In addition, 71% of the *OPA1* mutation carriers presented optic disc hyperemia, which has never been observed in typical patients with ADOA. By contrast, only 20% of those carriers showed temporal disc pallor, which is a prominent optic appearance in patients with ADOA (41–86%) (Cohn et al., 2007; Yu-Wai-Man et al., 2010; Chen et al., 2014). Four patients harboring the most common mutation c.2708_2711delTTAG had different fundus appearance. We speculate that the difference in the fundus performance may be due to the different course of vision loss of patients. In a previous study, Pradhan et al. described a 36-year old man who suffered a bilateral, painless visual loss during his anti-tuberculosis treatment that included EMB (Pradhan et al., 2010). This patient also had optic disc hyperemia and peripapillary hemorrhage. After a series of differential diagnoses, the researchers finally identified a nonsense p.R38X *OPA1* mutation in this patient and inferred that the visual loss in that patient had been exacerbated following EMB therapy. We speculate that optic disc hyperemia is an acute response to the toxic effect of EMB; consequently, a concomitant effect of *OPA1* mutations and EMB toxicity renders *OPA1* mutations carriers prone to optic disc hyperemia. This might be the reason why the *OPA1* mutations carriers presented more optic disc hyperemia than was observed in the patients without mutations, even though both groups had a similar disease time course. The mutation rate (38.3%) of *OPA1* mutations in this EON cohort was higher than that (9.6 and 7.6%) reported in a group of Chinese patients with suspected hereditary optic neuropathy (Chen et al., 2014) and other Han Chinese population (Zhang et al., 2017). One reason might be the patients harboring *OPA1* mutations are more sensitive to the toxicity of EMB and present more obvious visual defects as our mentioned above. Another reason might be due to the small number of patients in the current cohort.

In this cohort, we only identified four male patients carrying a LHON-mtDNA mutation, and this rate (8.5%) was much lower than the rate (38.3%) in patients harboring *OPA1* mutations and the rate (33%) in Chinese patients with LHON or suspected with LHON (Ji et al., 2008; Yu et al., 2010). Hwang et al. (2003) screened a cohort of 24 patients with EON for LHON-mtDNA mutations but were unable to identify any LHON-mtDNA mutations. Therefore, the existence of LHON-mtDNA mutations might be uncommon in patients with EON. To date, twelve patients (including our four patients) have been reported to develop optic neuropathy while taking EMB (Table 4; Dotti et al., 1998; De Marinis, 2001; Chowdhury et al., 2006; Ikeda et al., 2006; Seo et al., 2010). Ten of these patients carried mutation m.11778G > A, which is the most common primary mutation of LHON (Chen et al., 2014). In the present study, we described two patients carrying the m.14484T > C mutation (the second commonest primary mutation of LHON) who developed LHON while taking EMB (Table 4). Unlike the eight previously described cases, the four patients in our cohort had a younger onset age (48 years vs. 21.5 years). In addition, these four patients all had a family history, and three of them showed optic disc hyperemia and RNFL pseudoedema, which is a typical fundus appearance in the acute stage of LHON (Chen et al., 2014). Not all individuals who carry LHON-mtDNA mutations will develop visual symptoms, as the occurrence of LHON usually needs other risk factors, like heavy smoking and alcohol consumption (Sadun et al., 2003). EMB might have been a trigger or an epigenetic factor for the manifestation of LHON in these four patients.

**TABLE 4:** Clinical features of reported patients with EMB-induced LHON.

**ID**	**Onset age**	**Gender**	**Medication time**	**BCVA**	**Follow-up time**	**Follow-up BCVA**	**Optic disc finding**	**Mutation**	**References**
	**(years)**		**(months)**	**(OD/OS)**	**(months)**	**(OD/OS)**	**(OD/OS)**		
1	50–55	M	8	0.5/0.04	24	0.5/0.04	TEP/TEP	m.G11778A	Dotti et al., 1998
2	50–55	M	11	0.5/0.04	21	1.0/0.7	normal/normal	m.G11778A#	De Marinis, 2001
3	65–70	F	12	0.2/0.05	6	0.3/0.09	–	m.G11778A	Ikeda et al., 2006
4	65–70	F	3	0.03/0.03	12	0.01/0.01	normal/normal	m.G11778A	Ikeda et al., 2006
5	30–35	M	4	0.3/0.15	1	0.06/HM	H/H	m.G11778A	Chowdhury et al., 2006
6	40–45	M	4	0.1/0.1	5	FC/FC	normal/H	m.G11778A	Seo et al., 2010
7	20- 25	M	12	0.6/1.0	15	FC/FC	H/H	m.G11778A	Seo et al., 2010
8	30–35	F	4	FC/FC	-	-	TOP/TOP	m.G11778A	Seo et al., 2010
A2061	15–20	M	12	0.06/0.06	-	-	H/H	m.G11778A	This study
A2290	20–25	M	18	FC/FC	24	FC/FC	TEP/TEP	m.G11778A	This study
A781	15–20	M	12	0.8/0.02	–	–	H*/H	m.T14484C	This study
A1780	25–30	M	7	0.1/0.3	–	–	H/H	m.T14484C	This study

F, female; M, male; FC, finger counting; HM, hand move; TEP, temporal pallor; H, hyperemia; TOP, total pallor; *hemorrhage; #heteroplasmic.

The toxicity of EMB is related to its zinc-chelating effect and its metabolites (Chung et al., 2009). At present, the exact mechanism for the toxic neuropathy induced by EMB remains unclear, but increasing evidence indicates a relationship with mitochondrial dysfunction. In an early study, Heng et al. found that EMB was specifically toxic to retinal ganglion cells and that it caused ganglion cell degeneration by a glutamate excitotoxic pathway (Heng et al., 1999). LHON-mtDNA and *OPA1* mutations also cause damage to the small-caliber papillomacular bundle axons (Barboni et al., 2010) therefore, the clinical features of EON, LHON, and ADOA partially overlap. All three conditions show color vision abnormality and visual field defects. In the current cohort, most patients presented central or cecocentral scotoma, which could be observed in both EON and LHON or ADOA, whereas only three patients in group 2 showed temporal hemianopia, which is a typical visual field defect of EMB-related optic chiasmopathy (Jayanetti et al., 2017). In this cohort, the majority of patients presented with red/green color vision impairment or total color blindness, while only two patients carrying *OPA1* mutations displayed yellow/blue color vision impairment, which is a typical color defect of ADOA (Cohn et al., 2007). Fundus hemorrhage is not common in LHON or ADOA, and three of the four patients with fundus hemorrhage were in group 2, indicating it to be one of the EON clinical features. Our results showed that the mean age was significantly younger in the patients with mitochondrial mutations (27.5 years; group 1) than in the patients without mutations (48 years; group 2), further demonstrating that mitochondrial mutation was an important risk factor for the occurrence of EON, especially in young tuberculosis patients. Patients carrying mitochondrial mutations (*OPA1* or mtDNA) may be more vulnerable to the toxicity of EMB. Of the seven patients with a family history, six patients were mutation carriers; therefore, physicians should carefully question patients with tuberculosis or their family members about ophthalmic problems before EMB is prescribed.

Consistent with the observation by Lee et al. (2008) we found that the visual prognosis was related to the initial fundus appearance of the patients with EON. Up to 79.8% of the patients in our cohort showed normal optic disc appearance or optic disc hyperemia, which suggested they were in the early stage of EON. This might be a reason why our patients had a higher visual recovery rate (75.7%) than the rates described in other EON studies (23.1–47%) (Lee et al., 2008; Ezer et al., 2013). Another reason for this high rate might be due to that the 94.6% VA improvement was reported by patients subjectively; this is one limitation of the current study. Nevertheless, we still observed that the visual outcome was better in the patients in group 2 than in group 1, suggesting that the mitochondrial mutations were a critical factor affecting visual prognosis of patients with EON. Of the eight patients carrying mutation m.11778G > A, only one patient who harbored a heteroplasmic mutation showed VA improvement, (De Marinis, 2001) whereas the other patients all experienced VA decreases or no change during their follow-up (Table 4). Another limitation of the current study is that we did not take into account the ocular toxicity of other anti-tuberculosis drugs, such as isoniazid. The third limitation is that since the study was retrospective in nature, the examinations were not standardized.

In conclusion, our results indicated that carriers with *OPA1* mutations might be more vulnerable for the toxicity of EMB to develop EON, whereas the exact effect of these *OPA1* mutations has not been confirmed in future functional assays. Although mitochondrial mutation screening is not possible in all patients prior to anti-tuberculosis medication, genetic analysis should be strongly recommended for patients with a family history of optic neuropathy.

## Data Availability Statement

The data presented in the study are deposited in the NCBI GenBank, accession numbers NM_015560 and AC_000021.2.

## Ethics Statement

The studies involving human participants were reviewed and approved by Medical Ethics Committee of Beijing Tongren Hospital. The patients/participants provided their written informed consent to participate in this study.

## Author Contributions

X-HZ participated in the data collection, data analysis, and manuscript preparation. YX, KX, and KC participated in the data collection and analysis. Q-GX contributed in the study design and data collection. Z-BJ, YL, and S-HW participated in the study design and the manuscript revision. All authors contributed to the article and approved the submitted version.

## Conflict of Interest

The authors declare that the research was conducted in the absence of any commercial or financial relationships that could be construed as a potential conflict of interest.

## Publisher’s Note

All claims expressed in this article are solely those of the authors and do not necessarily represent those of their affiliated organizations, or those of the publisher, the editors and the reviewers. Any product that may be evaluated in this article, or claim that may be made by its manufacturer, is not guaranteed or endorsed by the publisher.

## Footnotes

1
https://www.mitomap.org

2
http://genetics.bwh.harvard.edu/pph2/

3
http://www.mutationtaster.org/

4
http://provean.jcvi.org/index.php

5
http://www.cbs.dtu.dk/services/NetGene2/

6
http://www.fruitfly.org/seq_tools/splice.html

7
http://browser.1000genomes.org/index.html

8
http://exac.broadinstitute.org/

## Supplementary Material

Supplementary Figure 1 Sanger sequencing electropherograms for 17 identified *OPA1* mutations in the study.

Supplementary Figure 2 Family pedigrees for seven patients with family history of optic neuropathy.
