# Supplementary figures and images for: Mitochondrial Mutations in Ethambutol-Induced Optic Neuropathy
Source: Front Cell Dev Biol. 2021 Oct 5;9:754676. doi: 10.3389/fcell.2021.754676 (PMC8525703; doi:10.3389/fcell.2021.754676)

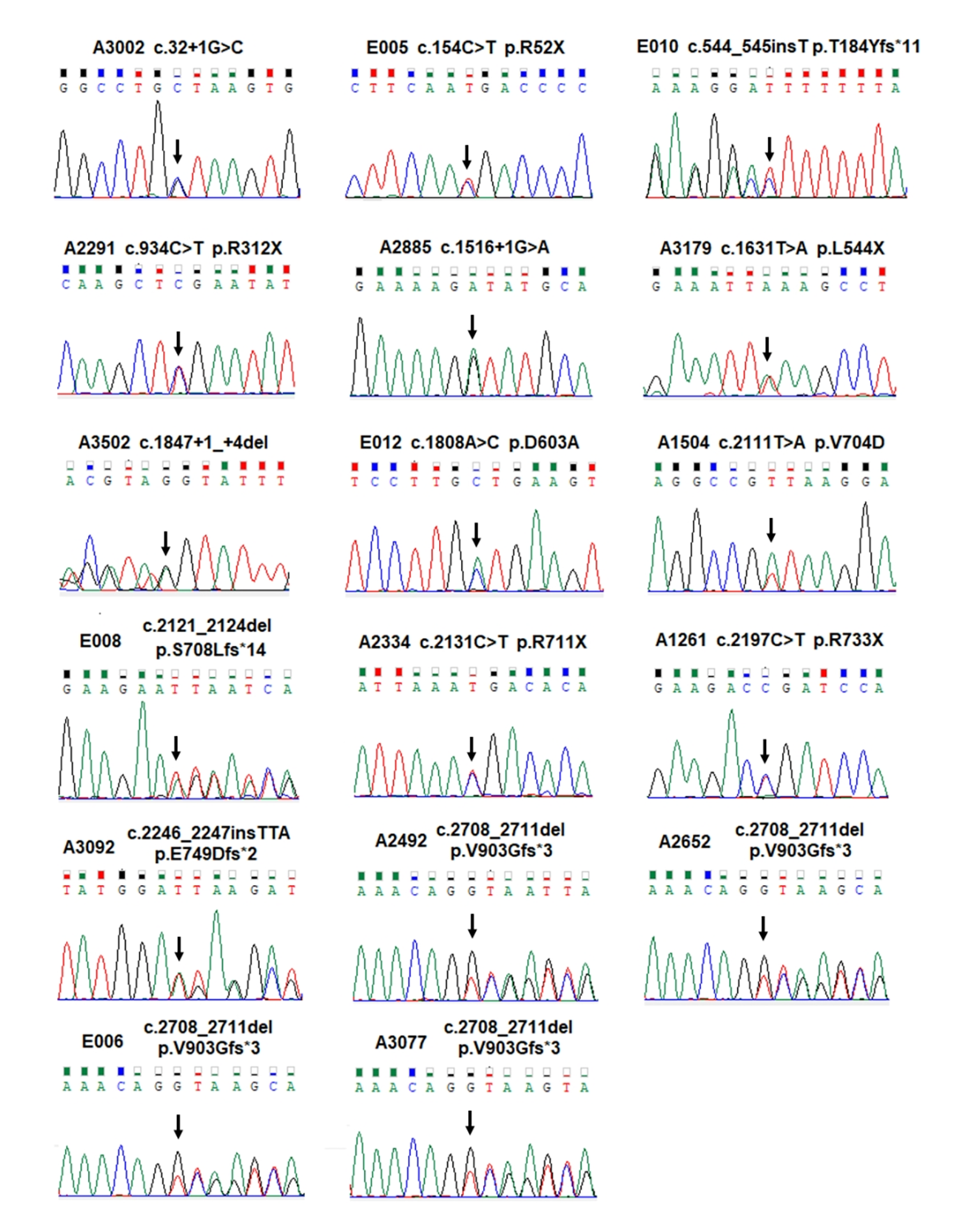

Supplement: Supplementary Figure 1 — Sanger sequencing electropherograms for 17 identified OPA1 mutations in the study. [file Image_1.TIF]

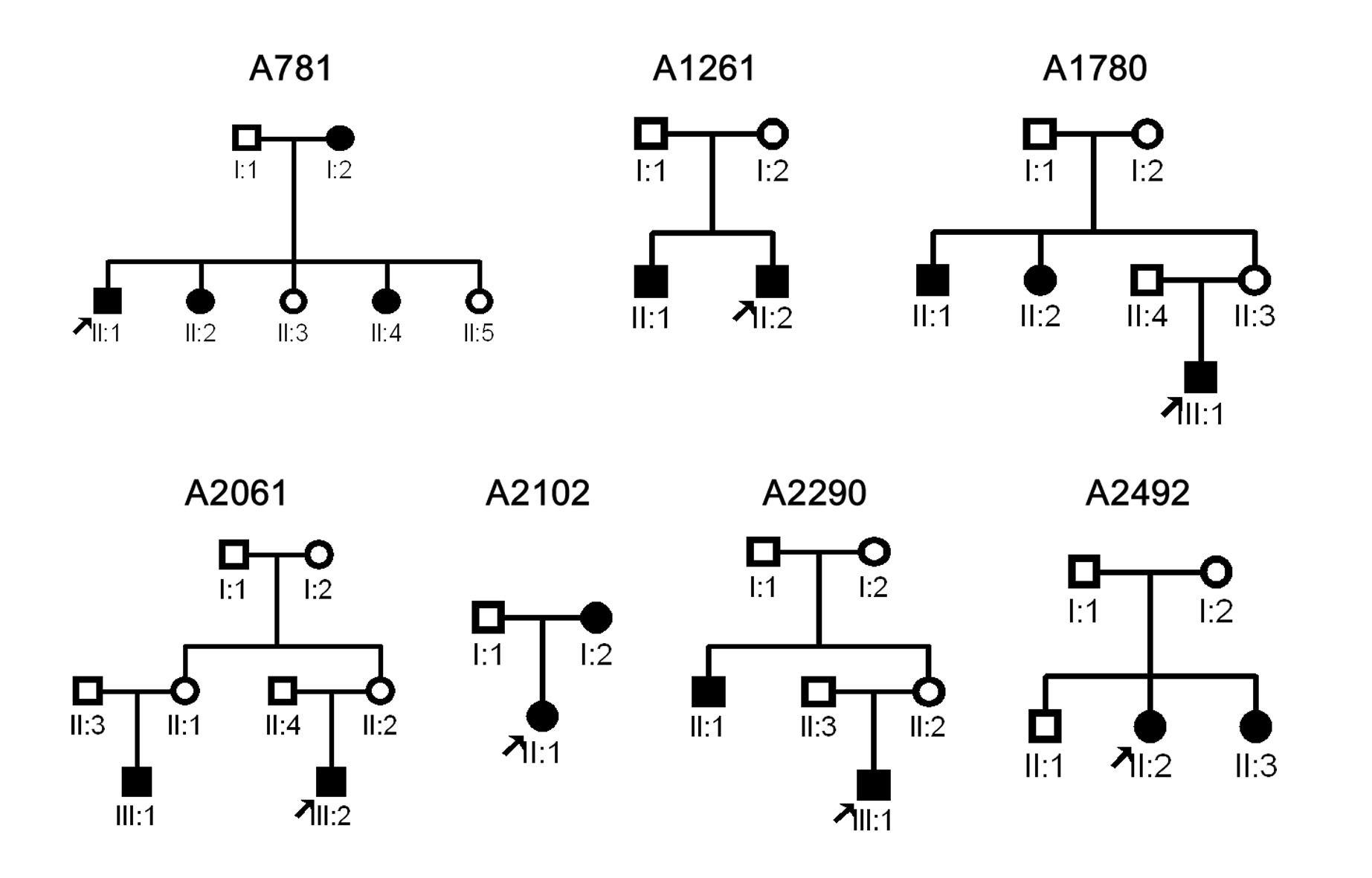

Supplement: Supplementary Figure 2 — Family pedigrees for seven patients with family history of optic neuropathy. [file Image_2.TIF]
