# Supplementary material for: Mitochondrial Mutations in Ethambutol-Induced Optic Neuropathy
Source: Front Cell Dev Biol. 2021 Oct 5;9:754676. doi: 10.3389/fcell.2021.754676 (PMC8525703; doi:10.3389/fcell.2021.754676)
Supplement: Supplementary file 3 [file Data_Sheet_1.PDF]

Supplementary table S1. The gene list of the capure panel in this study

Gene	Chromosome	Transcript ID	Capture length (bp)
ACO2	chr22	NM_001098	2725
ACTA1	chr1	NM_001100	1138
AFG3L2	chr18	NM_006796	3724
AIPL1	chr17	NM_014336	872
ANGPT1	chr8	NM_001146	1794
ANKRD34C-AS1	chr15	NR_038997	192
ANTXR1	chr2	NM_032208	2933
APTX	chr9	NM_001195249	1482
ARID1A	chr1	NM_006015	7552
ASB10	chr7	NM_001142459	1693
ATF6	chr1	NM_007348	2593
ATM	chr11	NM_000051	12210
ATP13A2	chr1	NM_001141974	4714
ATP1A3	chr19	NM_001256214	3670
ATXN1	chr6	NM_001357857	7589
ATXN10	chr22	NM_013236	1836
BFSP2	chr3	NM_003571	1439
C12orf57	chr12	NM_001301834	749
C12orf65	chr12	NM_001194995	499
CA11	chr19	NM_001217	2412
CACNA1A	chr19	NM_001127221	8219
CACNA1F	chrX	NM_005183	7508
CAPN3	chr15	NM_173087	4049
CASK	chrX	NM_003688	4098
CAV3	chr3	NM_033337	479
CC2D2A	chr4	NM_001080522	5786
CEP290	chr12	NM_025114	9169
CFL2	chr14	NM_138638	610
CISD2	chr4	NM_001008388	501
CLDN19	chr1	NM_001185117	968
CNGA3	chr2	NM_001298	2279
CNGB3	chr8	NM_019098	2984
COL25A1	chr4	NM_198721	5933
COL6A1	chr21	NM_001848	6334
COL6A2	chr21	NM_001849	6243
COL6A3	chr2	NM_004369	10984
CRB1	chr1	NM_201253	4367
CRX	chr19	NM_000554	899
CRYGC	chr2	NM_020989	652
DCN	chr12	NM_001920	1169
DGUOK-AS1	chr2	NR_104029	1384
DHCR7	chr11	NM_001360	1565
DNA2	chr10	NM_001080449	3559
DNM1L	chr12	NM_012062	3143
DUX4	chr10	NM_001363820	1756
DYSF	chr2	NM_001130983	8739
ELOVL4	chr6	NM_022726	1047
EMD	chrX	NM_000117	1005
EPHA2	chr1	NM_004431	3418
FHL1	chrX	NM_001159702	1067
FKRP	chr19	NM_024301	2370
FKTN	chr9	NM_006731	1680
FRMD7	chrX	NM_194277	2816

FTO	chr16	NM_001363894	2510
GDF6	chr8	NM_001001557	1265
GJA8	chr1	NM_005267	1311
GJB1	chrX	NM_000166	1902
GMPPB	chr3	NM_013334	990
GNAT2	chr1	NM_005272	1286
GP1BA	chr17	NM_000173	2094
GPR143	chrX	NM_000273	1868
GUCY2D	chr17	NM_000180	3724
HESX1	chr3	NM_003865	641
IMPDH1	chr7	NM_000883	2465
ITGA7	chr12	NM_002206	4260
KCNJ13	chr2	NM_002242	1081
KCTD7	chr7	NM_153033	1071
KIF21A	chr12	NM_001173464	6202
KLC2	chr11	NM_022822	2390
KLHL41	chr2	NM_006063	2070
KRT14	chr17	NM_000526	1644
KRT5	chr12	NM_000424	1999
LAMA2	chr6	NM_000426	10596
LARGE1	chr22	NM_004737	2509
LCA5	chr6	NM_181714	2145
LEMD2	chr6	NM_001348710	1980
LIM2	chr19	NM_030657	669
LRAT	chr4	NM_001301645	690
LRMDA	chr10	NM_001305581	1126
MANBA	chr4	NM_005908	2916
MAPT	chr17	NM_001123066	3324
MFN2	chr1	NM_001127660	2578
PJVK	chr2	NM_001042702	108
MRE11	chr11	NM_001330347	2869
MT-ATP6	chrM	-	681
MT-CO1	chrM	-	1542
MT-CO3	chrM	-	784
MT-CYB	chrM	-	1141
MT-ND1	chrM	-	956
MT-ND2	chrM	-	1042
MT-ND3	chrM	-	346
MT-ND4	chrM	-	1378
MT-ND4L	chrM	-	297
MT-ND5	chrM	-	1812
MT-ND6	chrM	-	525
MT-TI	chrM	-	69
MT-TL1	chrM	-	75
MT-TL2	chrM	-	71
MT-TN	chrM	-	73
MYH2	chr17	NM_017534	6474
MYH7	chr14	NM_000257	6842
MYOT	chr5	NM_006790	1205
NBAS	chr2	NM_015909	8380
NEB	chr2	NM_001164507	30042
NMNAT1	chr1	NM_001297778	1183
NR2F1	chr5	NM_005654	1349
NTF4	chr19	NM_006179	577
NYX	chrX	NM_022567	28277
OCA2	chr15	NM_001300984	3565
OPA1	chr3	NM_130833	4635

OPA3	chr19	NM_025136	938
OPN1LW	chrX	NM_020061	1144
OPN1MW3	chrX	NM_001330067	2288
OPTN	chr10	NM_021980	2207
PAX2	chr10	NM_001304569	1804
PAX6	chr11	NM_001368929	2343
PEX16	chr11	NM_004813	1670
PHOX2A	chr11	NM_005169	923
PJVK	chr2	NM_001042702	1983
PLA2G6	chr22	NM_001349864	5436
PMPCA	chr9	NM_015160	13104
POLG	chr15	NM_00112613	4092
POLG2	chr17	NM_007215	1590
POMGNT1	chr1	NM_017739	2883
POMT1	chr9	NM_001136114	2448
POMT2	chr14	NM_013382	3219
PRKCG	chr19	NM_002739	2672
PRPH2	chr6	NM_000322	1037
PRPS1	chrX	NM_002764	1077
RAB18	chr10	NM_001256411	1184
RAB3GAP1	chr2	NM_012233	4010
RAB3GAP2	chr1	NM_012414	5413
RAB7A	chr3	NM_004637	770
RD3	chr1	NM_183059	586
RDH12	chr14	NM_152443	1243
RNASEH1	chr2	NM_001286834	1141
ROBO3	chr11	NM_022370	3803
RPE65	chr1	NM_000329	2045
RPGRIP1	chr14	NM_020366	4631
RPGRIP1L	chr16	NM_001127897	4658
RRM2B	chr8	NM_001172477	1644
RS1	chrX	NM_000330	1086
RTN4IP1	chr6	NM_032730	2119
RYR1	chr19	NM_001042723	18414
SALL2	chr14	NM_005407	3273
SALL4	chr20	NM_020436	3450
SBF2	chr11	NM_030962	6336
SDHA	chr5	NM_001330758	2264
SELENON	chr1	NM_206926	1993
SGCA	chr17	NM_001135697	1454
SGCB	chr4	NM_000232	1064
SGCG	chr13	NM_000231	1058
SIX6	chr14	NM_007374	738
SLC16A2	chrX	NM_006517	1614
SLC24A1	chr15	NM_004727	3582
SLC24A5	chr15	NM_205850	1677
SLC25A1	chr22	NM_005984	2450
SLC25A15	chr13	NM_014252	999
SLC25A4	chr4	NM_001151	1001
SLC25A46	chr5	NM_001303250	5987
SLC38A8	chr16	NM_001080442	1594
SLC4A11	chr20	NM_001174090	3396
SLC6A5	chr11	NM_004211	2700
SLC9A6	chrX	NM_001042537	2641
SMCHD1	chr18	NM_015295	7945
SOX10	chr22	NM_006941	1397
SPATA7	chr14	NM_001040428	2468

SPG7	chr16	NM_001363850	2980
SYNE1	chr6	NM_001347702	27202
SYNE2	chr14	NM_182914	21867
TBC1D20	chr20	NM_144628	1379
TCAP	chr17	NM_003673	542
TEAD1	chr11	NM_021961	1632
TIMM8A	chrX	NM_004085	529
TK2	chr16	NM_001172645	1719
TLR6	chr4	NM_006068	2453
TMEM126A	chr11	NM_032273	671
TMEM67	chr8	NM_001142301	4762
TNNT1	chr19	NM_003283	1709
TPM3	chr1	NM_001278188	1380
TUBA8	chr22	NM_018943	1585
TUBB3	chr16	NM_001197181	1355
TUBGCP4	chr15	NM_014444	2646
TULP1	chr6	NM_003322	2302
TWNK	chr10	NM_001163813	2105
TYR	chr11	NM_000372	1733
TYRP1	chr9	NM_000550	1607
UCHL1	chr4	NM_004181	1243
VCAN	chr5	NM_004385	10345
WDR36	chr5	NM_139281	3709
WDR73	chr15	NM_032856	1482
WFS1	chr4	NM_001145853	3144
YME1L1	chr10	NM_014263	3033
ZFYVE26	chr14	NM_015346	10421
