# Supplementary material for: Mitochondrial Mutations in Ethambutol-Induced Optic Neuropathy
Source: Front Cell Dev Biol. 2021 Oct 5;9:754676. doi: 10.3389/fcell.2021.754676 (PMC8525703; doi:10.3389/fcell.2021.754676)
Supplement: Supplementary file 4 [file Data_Sheet_2.PDF]

Supplementary table S2. The clinical characteristic and mutation screening results of 47 patients with ethambutol-induced optic neuropathy

ID	Gender	Age (years)	Daily dose (mg/kg)	Medication time (months)	BCVA (OD/OS)	Course of the vision loss (months)	Visual outcome	Recovery time (months)	Color vision (OD/OS)	Visual field (OD/OS)	Optic disc appearance (OD/OS)	Family history of poor vision	Gene mutation	Cosegregation
A1261§	M	30 - 35	NA	2.5	0.04/0.05	1	NA	NA	UTC/UTC	CS/CS	H/H	Y	*OPA1* p.R733X	Y
A1504	M	15 - 20	12.5	3	0.15/0.1	0.5	unchanged	-	normal/normal	CS/CS	H/H	N	*OPA1* p.V704D	NA
A2291	F	25 - 30	16.0	3	0.3/0.2	1	improved	6	TCB/YB	PS/PS	H/H	N	*OPA1* p.R312X	Y
A2334	M	15 - 20	12.5	4	0.05/0.05	1	improved	7	TCB/TCB	CS/CS	H/H	N	*OPA1* p.R711X	NA
A2492	F	35 - 40	15.0	3	0.1/0.12	1	decreased	-	TCB/TCB	CS/CS	H/TEP	Y	*OPA1* p.V903Gfs*3	Y
A2652	M	25 - 30	9.4	8	0.5/0.2	4	NA	NA	YB/TCB	NA	H/normal	N	*OPA1* p.V903Gfs*3	Y
A2885	M	25 - 30	16.9	1.4	FC/FC	NA	improved	2	UTC/UTC	CCS/CCS	H/H	N	*OPA1* c.1516+1g>a	Y
A3002	M	50 - 55	12.5	3	0.06/0.05	3	unchanged	-	UTC/UTC	NA	TEP/TEP	N	*OPA1* c.32+1g>c	Y
A3077	M	45 - 50	NA	7	0.05/0.1	3	NA	NA	TCB/RG	CCS/CCS	normal/normal	N	*OPA1* p.V903Gfs*3	NA
A3092	M	45 - 50	8.3	7	0.25/0.2	2	improved	2	TCB/TCB	CCS/CCS	H/H	N	*OPA1* E749Dfs*2	Y
A3179	M	20 - 25	NA	3	0.02/0.02	2	NA	NA	UTC/UTC	NA	H,TEP/H,TEP	N	*OPA1* p.L544X	Y

A3502	F	50 - 55	12.9	22	0.07/FC	4	improved	6	TCB/TCB	CCS/CCS	TEP/TEP	N	*OPA1* c.1847+1_+4del GTAA	NA
E005§	M	40 - 45	3.8	2	0.02/0.04	2.3	improved	12	UTC/UTC	CCS/CS	H,TEP/H,TEP	N	*OPA1* p.R52X	Y
E006§	M	45 - 50	14.2	2.5	0.05/0.02	2	unchanged	-	NA	PS/CS	H/H	N	*OPA1* p.V903Gfs*3	NA
E008§	M	25 - 30	12.9	4	0.1/0.1	2	unchanged	-	NA	CS/CCS	H,TEP/H,TEP	N	*OPA1* p.S708Lfs*14	Y
E009§	M	15 - 20	15.0	3	0.05/0.05	2	improved	8	NA	CS/CS	H/H	N	*OPA1* exon12-17 deletion	NA
E010§	F	70 - 75	NA	3.5	0.3/0.12	4	NA	NA	NA	CCS/CCS	H/normal	N	*OPA1* p.T184yfs*11	NA
E012§	F	15 - 20	13.6	10	0.3/0.15	6	unchanged	-	NA	CS/CS	TEP/TEP	N	*OPA1* p.D603A	NA
A781	M	15 - 20	12.5	12	0.8/0.02	3	NA	NA	normal/RG	NA	H*/H	Y	m.T14484C	Y
A1780	M	25 - 30	NA	7	0.1/0.3	1	NA	NA	YB/RG	CS/PS	H/H	Y	m.T14484C	NA
A2061	M	15 - 20	NA	12	0.06/0.06	2	NA	NA	RG/RG	CS/CS	H/H	Y	m.G11778A	NA
A2290	M	20 - 25	14.2	18	FC/FC	10	unchanged	-	RG/RG	NA	TEP/TEP	Y	m.G11778A	NA
A1242	M	20 - 25	12.9	4	0.7/0.2	1	improved	3	normal/YB	CS/CS	H/H	N	N	-
A1601§	M	55 - 60	10.2	24	0.02/0.01	3	improved	12	RG/UTC	TH/TH	normal/normal	N	N	-

A1866	M	30 - 35	NA	1	0.3/NLP	1	NA	NA	normal/UTC	NA	H/H,TEP	N	N	-
A2102	F	35 - 40	NA	3	0.4/0.6	24	NA	NA	YB/TCB	CCS/CCS	TOP/TOP#	Y	N	-
A2341	F	15 - 20	13.6	2	0.05/0.05	2	improved	1	RG/TCB	CS/CS	TEP/TEP	N	N	-
A2460	M	35 - 40	8.8	5	0.8/0.3	2	improved	1	normal/TCB	NA	H/H	N	N	-
A2483	F	70 - 75	15.6	8	0.12/0.06	2	improved	6	UTC/UTC	PS/CS	normal/normal	N	N	-
A2563	F	40 - 45	13.6	7	0.6/0.6	1	improved	6	TCB/YB	CS/PS	H/H	N	N	-
A2579	F	65 - 70	15.0	4	0.2/0.05	2	improved	6	RG/RG	CS/CS	normal*/normal	N	N	-
A2647	M	45 - 50	10.7	5	FC/FC	4	unchanged	-	RG/RG	NA	TOP/TOP	N	N	-
A2694	M	20 - 25	10.6	2.5	0.1/0.1	1	improved	3	NA	CCS/CCS	H/H	N	N	-
A2706	F	55 - 60	12.9	6	0.15/0.02	3	improved	2	RG/RG	CCS/CCS	H/H	N	N	-
A2870	F	30 - 35	13.4	12	0.06/0.02	2	improved	4	RG/UTC	CCS/CCS	H/H	N	N	-
A3124	F	60 - 65	11.5	3	0.05/0.03	2	improved	3	UTC/UTC	NA	H/H	N	N	-
A3129	F	50 - 55	13.6	1.5	0.01/FC	2	improved	5	RG/RG	NA	normal/normal	N	N	-

A3198	M	25 - 30	12.1	7	0.05/0.01	1	improved	3	TCB/TCB	NA	normal/normal[*]	N	N	-
A3268	M	45 - 50	15.4	3	0.15/0.15	3	improved	1	RG/RG	CCS/CCS	H/H	N	N	-
A3310	M	45 - 50	11.0	2	0.03/0.1	2	improved	9	YB/TCB	NA	H/H	N	N	-
A3332	F	50 - 55	15.3	5.5	0.1/0.1	3	improved	1.5	RG/TCB	CCS/CS	H/H	N	N	-
A3348	M	55 - 60	14.4	6.5	0.04/FC	1	improved	3	UTC/UTC	NA	normal/normal	N	N	-
A3371	M	20 - 25	15.3	8	0.12/0.05	0.5	improved	3	YB/UTC	NA	H,TEP/H,TEP	N	N	-
A3385	F	70 - 75	12.5	18	0.5/FC	5	improved	6	TCB/UTC	TH/TH	normal/normal	N	N	-
E001§	M	40 - 45	11.5	10	0.3/0.6	5	unchanged	-	normal/normal	TH/TH	TEP/TEP	N	N	-
E002§	M	55 - 60	29.2	6	0.08/0.08	1	improved	1	TCB/TCB	CS/CS	normal/normal	N	N	-
E004§	M	25 - 30	11.0	4	0.7/0.8	0.7	improved	4	RG/RG	CS/CS	TEP/TEP[*]	N	N	-

Note: F, female; M, male; Y, yes; N, no; FC, finger counting; NLP, no light perception; OD, right eye; OS, left eye; NA, not available; UTC, unable to cooperate; YB, yellow/blue color impairment; TCB, total color blindness; RG, red/green color vision impairment; CS, central scotoma; PS, paracentral scotoma; CCS, cecocentral scotoma;TH, temporal hemianopia; TEP, temporal pallor; H, hyperemia; TOP, total pallor; * fundus hemorrhage; # retinal nerve fiber layer edema; § the pathents with TES analysis.
