# Supplementary material for: Mitochondrial Mutations in Ethambutol-Induced Optic Neuropathy
Source: Front Cell Dev Biol. 2021 Oct 5;9:754676. doi: 10.3389/fcell.2021.754676 (PMC8525703; doi:10.3389/fcell.2021.754676)
Supplement: Supplementary file 5 [file Data_Sheet_3.PDF]

Supplementary table S3. Association between visual outcome and other variables

Variables		Coefficients	SE	95%CI	P value
Intercept		1.043	0.077		0.047
Course		-0.040	0.026	-0.091, 0.011	0.122
Optic Disc	HM	0.042	0.108	-0.170, 0.254	0.695
Optic Disc	P	-0.338	0.115	-0.563, -0.113	0.004
Optic Disc	H	Reference	.	.	.
Gene Mutation	Y	-0.351	0.091	-0.529, -0.173	<0.001
Gene Mutation	N	Reference	.	.	.
